# Gain-of-function mutations in Aqp3a influence zebrafish pigment pattern formation through the tissue environment

**DOI:** 10.1242/dev.143495

**Published:** 2017-06-01

**Authors:** Anastasia Eskova, Francois Chauvigné, Hans-Martin Maischein, Moritz Ammelburg, Joan Cerdà, Christiane Nüsslein-Volhard, Uwe Irion

**Affiliations:** 1 Max Planck Institute for Developmental Biology, 72076 Tübingen, Germany; 2 IRTA-Institut de Ciències del Mar, Consejo Superior de Investigaciones Científicas (CSIC), 08003 Barcelona, Spain

**Keywords:** Aquaporin, Pattern formation, Pigmentation, Zebrafish

## Abstract

The development of the pigmentation pattern in zebrafish is a tightly regulated process that depends on both the self-organizing properties of pigment cells and extrinsic cues from other tissues. Many of the known mutations that alter the pattern act cell-autonomously in pigment cells, and our knowledge about external regulators is limited. Here, we describe novel zebrafish *mau* mutants, which encompass several dominant missense mutations in Aquaporin 3a (Aqp3a) that lead to broken stripes and short fins. A loss-of-function *aqp3a* allele, generated by CRISPR-Cas9, has no phenotypic consequences, demonstrating that Aqp3a is dispensable for normal development. Strikingly, the pigment cells from dominant *mau* mutants are capable of forming a wild-type pattern when developing in a wild-type environment, but the surrounding tissues in the mutants influence pigment cell behaviour and interfere with the patterning process. The mutated amino acid residues in the dominant alleles line the pore surface of Aqp3a and influence pore permeability. These results demonstrate an important effect of the tissue environment on pigment cell behaviour and, thereby, on pattern formation.

## Abstract (precis)

**Summary:** Dominant mutations in the water channel Aquaporin 3a cause defective pigment patterning in zebrafish, due at least in part to an effect of the mutant tissue environment on the pigment cells.

## INTRODUCTION

The zebrafish adult colour pattern of alternating blue and gold stripes is the result of spatially and temporarily controlled interactions among pigment cells (chromatophores): yellow xanthophores, silvery iridophores and black melanophores. In the light stripes, tightly packed light-reflective iridophores are covered by a layer of compact xanthophores (Hirata et al., 2003, 2005). Melanophores are confined to the dark stripes, where they are overlaid by a network of blue iridophores and loose xanthophores. This pattern arises during metamorphosis (3-6 weeks post fertilization), when iridophores migrate into the skin to form the first light stripe along the horizontal myoseptum. Next, metamorphic melanophores appear in the prospective regions of the first two dark stripes. At the edges of the light stripe, the iridophores disperse and migrate dorsoventrally into the adjacent dark stripe regions. Then, upon reaching the positions of the next light stripes, they become tightly packed again. At this point, xanthophores evenly cover the skin, increasing in density in response to tightly packed iridophores and dispersing in response to melanophores (Mahalwar et al., 2014). The whole process is repeated to form the next stripes. The adult zebrafish trunk can contain up to five dark stripes interspersed with light stripes (Singh et al., 2014, 2016).

The formation of the pattern requires the presence of all three types of chromatophores and their mutual interactions. A number of regulators of pigment cell differentiation, survival and behaviour are known (reviewed by Singh and Nüsslein-Volhard, 2015). Many are transcription factors or signalling molecules involved in cell specification or survival and thus affect the pigment cells or their precursors directly. Others, such as ion channels, connexins and Tight junction protein 1A, are required for cell-cell communications, which are crucial for the spatial arrangement of chromatophores and the cell shape transitions of iridophores and xanthophores (Maderspacher and Nüsslein-Volhard, 2003; Watanabe et al., 2006; Irion et al., 2014; Fadeev et al., 2015; Mahalwar et al., 2016).

Pattern formation cannot be attributed entirely to self-organizing properties of the pigment cells, and not all regions of the body are patterned equally. In fact, the dorsal- and ventral-most parts lack stripes, despite the presence of pigment cells. Very little is known about the role of the external milieu in determining pigment cell behaviour. Extrinsic regulators of stripe formation include the horizontal myoseptum, a general anatomical pre-pattern that directs where iridophores initially emerge in the skin to form the first light stripe (Singh et al., 2014). In addition, external ligands can locally activate receptors expressed in pigment cells, e.g. Csf1r in xanthophores or Ednrb1a (Ednrba) in iridophores (Krauss et al., 2014; Parichy et al., 2000b). Endocrine regulation also plays a role: thyroid hormone promotes xanthophore proliferation and represses melanophores (McMenamin et al., 2014). Finally, the communication between chromatophores via gap junctions and potassium channels may be regulated by the local tissue environment. Their conductivity depends on the polyamine spermidine (Ficker et al., 1994; Watanabe et al., 2012), and a mutation in the gene encoding spermidine synthase affects stripe width (Frohnhöfer et al., 2016).

Here, we describe zebrafish *mau* mutants, which have gain-of-function mutations in the gene encoding Aquaporin 3a (Aqp3a) that affect pigment patterning and fin growth. Aquaporins are tetrameric pore proteins that facilitate water diffusion through biological membranes while excluding ions. One subgroup, the aqua-glyceroporins, can also transport non-polar solutes, such as glycerol, peroxide and urea. Some aquaporins can be permeable for other unconventional solutes such as ammonia and hydrogen peroxide, as well as some anions (Wu and Beitz, 2007). The zebrafish genome encodes a large repertoire of aquaporins, many of which show tissue-specific expression (Tingaud-Sequeira et al., 2010).

Point mutations in *aqp3a* result in a dominant phenotype with broken, undulating stripes and short fins. The most severely affected pigment cells in *mau* mutants are larval xanthophores, which deteriorate before metamorphosis and only partially recover during later development. We identified four alleles with altered amino acid residues that line the pore surface of Aqp3a and change pore permeability. A loss-of-function allele, generated by the CRISPR-Cas9 system, has no phenotypic consequences, indicating that Aqp3a is dispensable for normal development. In contrast to other zebrafish mutants with spotted patterns, namely *leo*, *luc*, *sbr* or *seurat* (Maderspacher and Nüsslein-Volhard, 2003; Irion et al., 2014; Fadeev et al., 2015; Eom et al., 2012), all three types of pigment cells from dominant *mau* mutants are capable of forming a wild-type pattern when surrounded by wild-type tissues, but wild-type pigment cells fail to do so when surrounded by mutant tissues. Our results emphasize the importance of the tissue environment in regulating pigment cell behaviour during pattern formation.

## RESULTS

### *mau* mutants have missense mutations in *aqp3a*

**Fig. 1.**
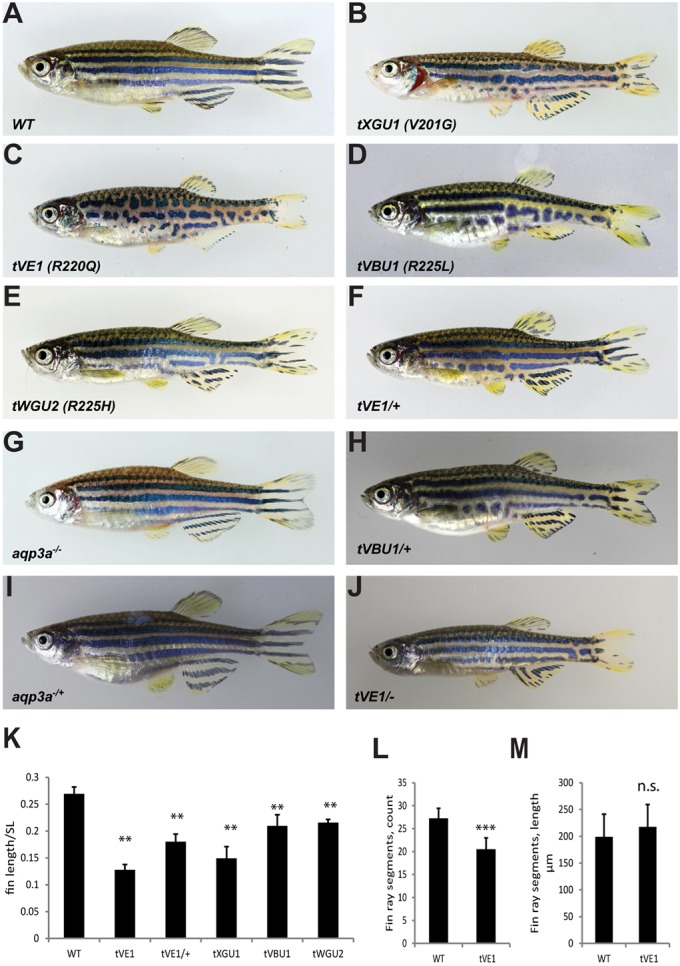
**Mutations in *aqp3a* lead to undulating, broken stripes and short fins.** (A-E) Comparison of wild type with *mau* mutants carrying the dominant alleles *tVE1*, *tXGU1*, *tVBU1* and *tWGU2*. (F,H) The dominant *mau* phenotype is attenuated in heterozygous fish. (G,I) The loss-of-function mutant *aqp3a*^−/−^, generated in the background of *aqp3a^tVE1/tVE1^*, has a wild-type phenotype in homozygous and heterozygous fish. (J) The *aqp3a^tVE1/−^* phenotype is similar to that of *aqp3a^tVE1/+^*. (K) Ratio of caudal fin length to standard body length (SL) in *mau* mutants. For each allele, seven to nine fish were measured. Mean±s.d. **P<0.01 for mutant versus wild type (one-way ANOVA followed by Tukey-Kramer multiple comparisons test). (L) Number of fin ray segments in the third ray of the caudal fin in wild-type and *aqp3a^t^^VE1/tVE1^* fish. *n*=8. Mean±s.d. ***P<0.0001 (unpaired *t*-test). (M) Length of fin ray segments in the third ray of the caudal fin in wild-type and *aqp3a^t^^VE1/tVE1^* fish. *n*=8. Mean±s.d. n.s., not significant (unpaired *t*-test).

**Fig. 2.**
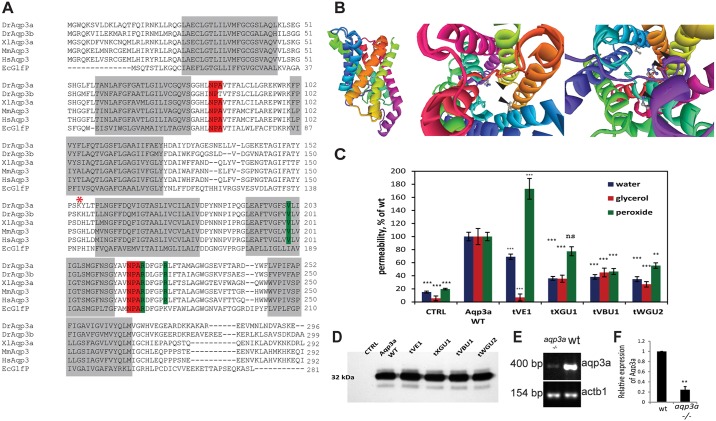
**Mutations in *aqp3a* alter pore permeability.** (A) Alignment of Aqp3a amino acid sequence with zebrafish paralogue Aqp3b and homologues from human (Hs), mouse (Mm), *X. laevis* (Xl), and *E. coli* (Ec). The transmembrane domains and the conserved NPA motifs of the water selectivity filter are highlighted in grey and red, respectively. Mutated amino acid residues in the dominant *mau* alleles are highlighted in green. The start of the frameshift in the loss-of-function allele is marked with an asterisk. (B) Homology-based model of zebrafish Aqp3a. (Left) Side view of the aquaporin monomer. (Middle) View from the cytoplasmic side of the channel, emphasizing mutated amino acids. (Right) View from the extracellular side of the channel. Arrowheads point to the mutated amino acids in the pore surface of the protein. (C) Mutant variants of Aqp3a have altered permeability for water, glycerol and peroxide when expressed in *Xenopus* oocytes. CTRL, uninjected oocytes. Solute and allele data were obtained from 11-12 oocytes and normalized to wild type. Mean±s.e.m. ***P<0.001, **P<0.01; ns, not statistically significant; wild-type and mutant aquaporin variants versus uninjected oocytes for each cargo (unpaired *t*-test). (D) Western blot showing expression levels of wild-type and mutant zebrafish Aqp3a in *Xenopus* oocytes. CTRL, water-injected oocytes. Total membranes from ten oocytes were pooled and equivalent membrane extract from two oocytes was loaded on the gel. (E) Semi-quantitative RT-PCR showing reduced abundance of *aqp3a* transcript in *aqp3a*^−/−^ compared with the wild-type sibling. *actb1* was used as a control. (F) The intensity of the *aqp3a* band in E was normalized to the *actb1* control to compare wild type and *aqp3a*^−/−^. *n*=3. **P<0.01, paired *t*-test.

In several screens for zebrafish mutants with pigment pattern defects we identified four *mau* mutant alleles (*tVE1*, *tXGU1*, *tVBU1*, *tWGU2*), which exhibited similar phenotypes with undulating, broken stripes and short fins (Fig. 1A-E,K-M). The mutations are dominant, with heterozygous fish showing a similar but weaker phenotype (Fig. 1F,H). The mutations were mapped to a 3.7 Mb region on chromosome 5 between markers z9568 and z13641. Because this region shows a very low frequency of sequence polymorphisms among our mapping strains, we performed whole-genome sequencing of fish homozygous for the allele *tVE1*. The only candidate for a phenotype-causing mutation in the entire region was a missense mutation in exon 5 of the *aqp3a* gene. Sequencing of *aqp3a* in the other three *mau* mutant alleles also identified missense mutations (Table 1, Fig. 2A).

**Table 1.**
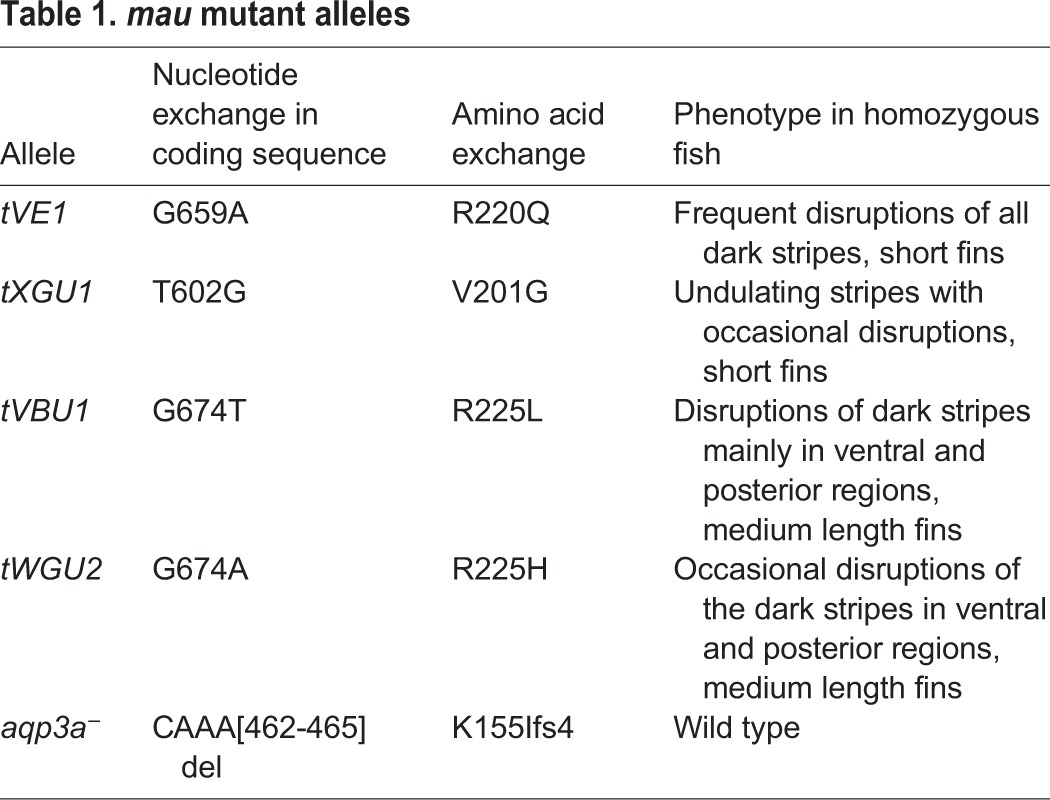
***mau* mutant alleles**

The mutations affect conserved amino acid residues of Aqp3a: R220, which is mutated in the strongest allele *tVE1*, invariably follows the NPA motif that forms the water selectivity filter (Sui et al., 2001); V201, which is mutated in *tXGU1*, and R225, which is mutated in *tVBU1* and *tWGU2*, are conserved among aqua-glyceroporins (Fig. 2A) (Tingaud-Sequeira et al., 2010).

### The pore permeability of Aqp3a is affected in *mau* mutants

We modelled the zebrafish Aqp3a protein structure based on the known structure of the *E.coli* glycerol transporter GlpF, which is homologous to animal aqua-glyceroporins (Fu et al., 2000; Tajkhorshid et al., 2002). The model predicts that all of the affected amino acids line the pore surface (Fig. 2B) and are thus likely to affect pore permeability. To assess this, we tested the permeability for water, glycerol and peroxide, which are known cargos of Aqp3 (Ma et al., 1994; Echevarria et al., 1994; Miller et al., 2010; Tingaud-Sequeira et al., 2010), of wild-type and mutant zebrafish Aqp3a in *Xenopus* oocytes. Permeability for water and glycerol was significantly reduced in all mutants, whereas peroxide permeability increased by 80% in *tVE1*, but was reduced in the other alleles (Fig. 2C,D). Taken together, these results suggest that the structure and functionality of the Aqp3a channel are altered in the dominant *mau* mutants.

### The *aqp3a* mutations of *mau* mutants are neomorphic and dose dependent

To test if the dominance observed in *mau* mutants is due to haploinsufficiency or caused by a gain-of-function, we used the CRISPR-Cas9 system (Hwang et al., 2013) to target *aqp3a* exon 4, which is upstream of the identified point mutations. A 4 bp deletion induced in *mau* embryos homozygous for the *tVE1* allele leads to complete reversion of the phenotype, giving rise to heterozygous and homozygous fish of wild-type appearance (Fig. 1G,I). This deletion is predicted to be a null allele, as it causes a frameshift followed by a premature stop codon resulting in a truncated 158 amino acid polypeptide that lacks the last three transmembrane domains. The mutation leads to the downregulation of the corresponding transcript (Fig. 2E,F), possibly owing to nonsense-mediated mRNA decay. A comparable truncation, caused by exon 5 skipping, has been reported in humans where it leads to a complete loss of AQP3 in red blood cells (Roudier et al., 2002).

**Fig. 3.**
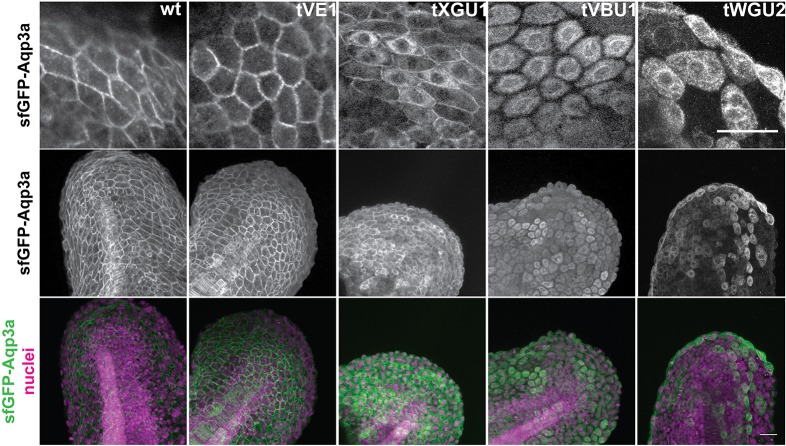
**GFP-Aqp3a localization in zebrafish fin fold cells.** mRNA of *GFP-Aq3a* was microinjected at the 1-cell stage and the protein localization was imaged at 1dpf. Wild-type and Aqp3aR220Q (tVE1) proteins reach the plasma membrane, whereas Aqp3aR225L (tVBU1) and Aqp3aR225H (tWGU2) mutants are retained intracellularly. Aqp3aV201G (tXGU1) shows partial retention. Nuclei are stained with Hoechst 33342. Scale bars: 25 µm.

To test whether the point mutations in Aqp3a might affect protein folding, we fused wild-type and mutant proteins to Strep-tag and super-folded GFP (sfGFP). When expressed in HeLa cells (Fig. S1A,B) or in the fin fold of zebrafish embryos (Fig. 3), wild-type and tVE1 fusion proteins localized to the plasma membrane; however, the remaining three mutant alleles showed either partial (tXGU1) or strong (VBU1 and WGU2) retention in the endoplasmic reticulum (ER). Interestingly, the amount of the mutant protein delivered to the plasma membrane, bypassing ER retention, appeared to correlate with the strength of the *mau* phenotype.

Together with the observations that heterozygous *mau* mutants have a weaker phenotype than homozygotes, and that the phenotype of *aqp3a^tVE1/−^* fish is similar to that of *aqp3a^tVE1/+^* (Fig. 1F,J), these data suggest that the *aqp3a* mutations in *mau* mutants are neomorphic and act dominantly in a dose-dependent manner.

### Chromatophores are affected indirectly in *mau* mutants

**Fig. 4.**
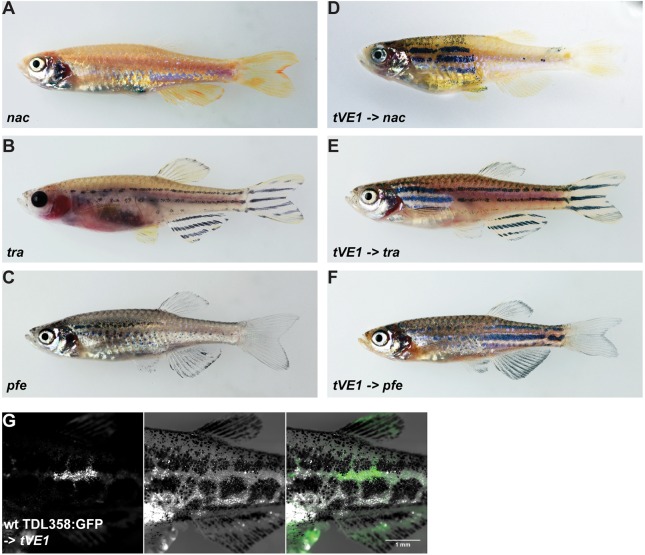
**Dominant mutations of** ***aqp3a* do not act cell-autonomously in pigment cells.** (A-C) Blastomere transplantations of *aqp3a*^*tVE1/tVE1*^ into mutants lacking one chromatophore type (*nacre*, *transparent* or *pfeffer*). (D-F) Typical chimeric animals resulting from transplantations show the striped wild-type pattern in the regions where *mau* pigment cells complement the host residual pattern. (G) Wild-type iridophores *Tg(TDL358:GFP)* do not form a wild-type pattern when transplanted into *aqp3a^tVE1/tVE1^*. Left: GFP-labeled iridophores; middle: corresponding bright-field image; right: merged image.

To test whether the patterning defects in *mau* mutants are caused by pigment cells, we transplanted *mau* mutant blastomeres into embryos of *nacre* (*mitfa*), *pfeffer* (*csf1ra*) and *transparent* (*mpv17*) mutants, which lack melanophores, xanthophores and iridophores, respectively (Lister et al., 1999; Odenthal et al., 1996; Krauss et al., 2013) (Fig. 4A-C). In all three cases, mutant pigment cells contributed to the wild-type pattern in the resulting chimeric animals (Fig. 4D-F), indicating that the *mau* phenotype is not based on mutant pigment cells alone. By contrast, when wild-type blastomeres carrying *Tg(TDL358:GFP)*-labelled iridophores were transplanted into *mau* mutant embryos, they failed to form proper stripes (Fig. 4G). This suggests that although *mau* pigment cells are normal and capable of forming the wild-type pattern, the mutant tissue environment prevents them from doing so.

### Development of the *mau* phenotype

**Fig. 5.**
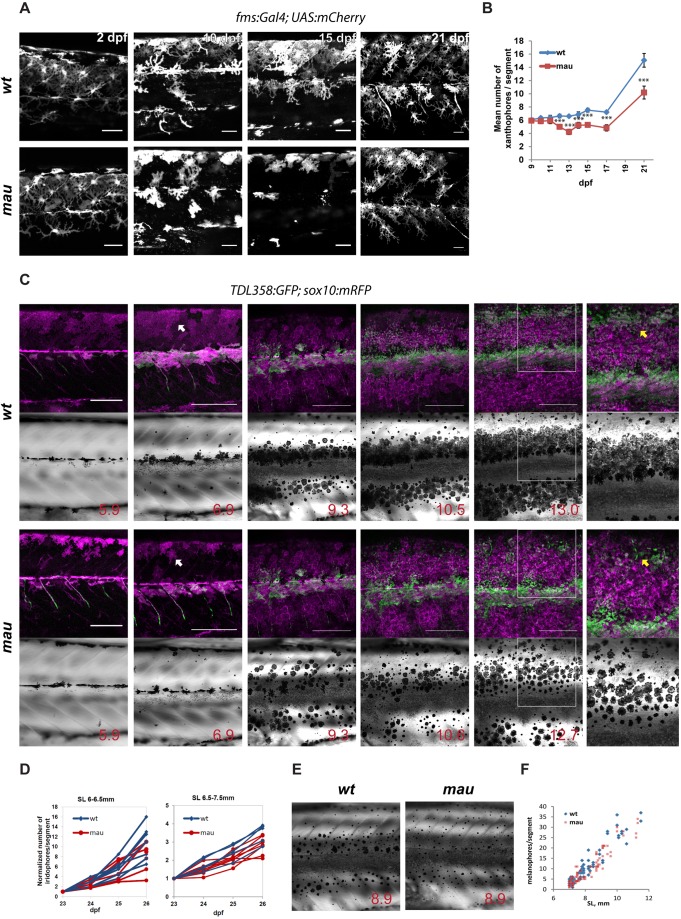
**Development of the** ***mau*** **phenotype in *aqp3a^tVE1/tVE1^*.** (A) Xanthophores labelled by *Tg(fms:GAL4; UAS:mCherry)* in wild-type and in *aqp3a^tVE1/tVE1^* (*mau*) larvae. By 15 dpf, the numbers and the area covered by xanthophores are reduced in the mutants, but xanthophores later recover. (B) Numbers of xanthophores per segment in the mid-trunk of pre-metamorphic fish (*n*=8-10 fish); data points represent mean±s.e.m. ***P<0.0001, unpaired *t*-test. (C) Representative example of recurrent imaging of individual fish carrying *Tg(TDL358:GFP)* (green, labelling iridophores) and *Tg(sox10:mRFP)* (magenta, labelling all the pigment cells) in wild type and in *mau*. Standard length (SL) is indicated. White arrows point to contracted xanthophores [labelled in *mau*, *Tg(sox10:mRFP)*], whereas the wild-type xanthophores are spread and cover the entire flank of the fish. Yellow arrows indicate the position of the second light stripe. In *mau*, iridophores are present in the area but fail to form a continuous light stripe. (D) Increase in iridophore numbers per segment over the course of 4 days. The quantifications were obtained from recurrent imaging in the mid-trunk segments of the fish during the initial formation of the light stripe (SL 6-6.5 mm), and upon completion of the first light stripe (SL 6.5-7.5 mm). (E) *mau* melanophores are scattered and not confined to the dark stripe region. (F) Numbers of flank melanophores per segment in the mid-trunk (two segments above the anus) imaged in metamorphic wild-type and *mau* fish. Each data point represents the number of melanophores in one segment. Scale bars: 50 µm in A; 250 µm in C.

The early larval pigment pattern of *mau* mutants appears similar to wild type (data not shown). To understand the changes in pigment cell development and behaviour that create the *mau* pattern, we followed the development of homozygous *tVE1* fish carrying markers for the different pigment cells. The first pigment cells that will later contribute to the adult pattern are the larval xanthophores (Mahalwar et al., 2014). At 2 days post fertilization (dpf ) they cover the entire flank of *mau* fish, similar to wild type. In wild type the larval xanthophores persist and begin to proliferate at the onset of metamorphosis (Mahalwar et al., 2014); however, in *mau*, the xanthophores lose contact with each other (visible at 10 dpf), begin to deteriorate, and by 15 dpf mostly disappear from the flank. Later on, they partially recover and persist to adulthood (Fig. 5A,B).

During early metamorphosis, iridophores are similar in number and appearance in *mau* and wild-type fish [Fig. 5C, standard length (SL) 5.9 mm]. Later, the light stripe boundaries in *mau* are often less well defined compared with wild type (Fig. 5C, SL 6.9 mm). The proliferation rates of iridophores are also comparable between wild type and *mau* (Fig. 5D). Clear differences become apparent by the time the second dorsal light stripe is formed. Melanophores are present in normal numbers (Fig. 5F) in *mau*, and their appearance and density are normal; however, they are more dispersed (Fig. 5C, SL 9.3, 10.6 and 12.7-13 mm; Fig. 5E). Dense iridophores do not adopt a contiguous arrangement in the region of the second light stripe, even though patches of dense iridophores are present in this area (Fig. 5C, SL 12.7-13 mm).

Although all three types of chromatophores initially appear normal, the sharpening of dark and light stripes is affected. Transient loss and recovery of larval xanthophores is the most striking difference in chromatophore development between *mau* and wild type.

### Effect of the tissue environment on the behaviour of pigment cells

**Fig. 6.**
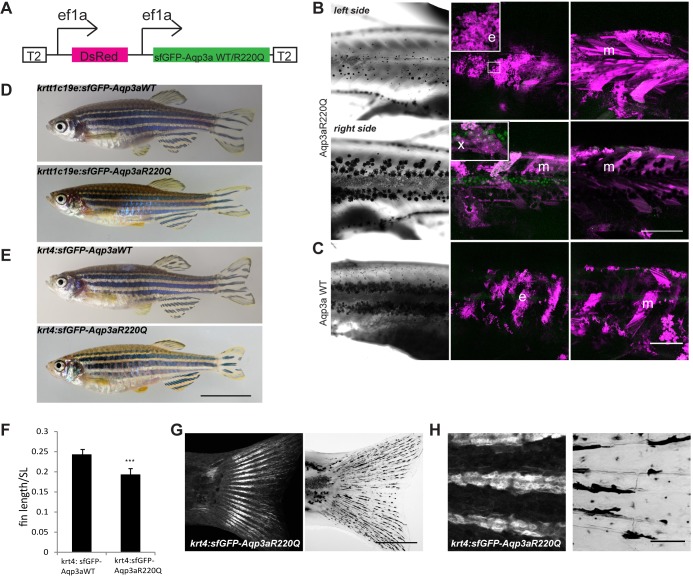
**Misexpression of wild-type (WT) and R220Q (tVE1) Aqp3a.** (A) Construct used for the analysis of mosaic expression. Ubiquitous *ef1a* promoters drive the simultaneous expression of dsRed and sfGFP-Aqp3aR220Q. The construct is flanked by Tol2 repeats (T2) for integration in the genome. (B) Mosaic expression of the *ef1a:DsRed, ef1a:sfGFP-Aqp3aR220Q* on two sides of the same fish. The middle panel shows maximum intensity projections of clones in the epidermis (35-40 µm) (e) and xanthophore clones in the hypodermis (x). The right panel shows maximum intensity projections of muscle clones (m) (40-100 µm). The loss of iridophores and scattering of melanophores occur on the left side, where the large epidermal clone coincides with a large muscle clone. A large muscle clone alone or clones in xanthophores do not produce any effect on pigment cell organization on the right-hand side of the fish. (C) Expression of wild-type Aqp3a does not affect pigment patterning. (D,E) Transgenic fish expressing sfGFP-Aqp3a WT or R220Q under the basal epidermis-specific promoter *krtt1c19e* (D) and under the *krt4* promoter (E). (F) Ratio of caudal fin length to standard body length (SL) in *Tg(krt4:sfGFP-Aqp3a WT)* and *Tg(krt4:sfGFP-Aqp3aR220Q)* fish. For each line, five to seven fish were measured. Mean±s.d. ***P<0.001, unpaired *t*-test. (G) *Tg(krt4:sfGFP-Aqp3aR220Q)* is expressed in fins of metamorphic fish. (H) The expression of *krt4:sfGFP-Aqp3aR220Q* is prominent in the cells of fin rays. Scale bars: 1 cm in E; 500 µm in B,C,G; 50 µm in H.

Aqp3a is expressed in most tissues in zebrafish, including skin and muscle (Hamdi et al., 2009; Tingaud-Sequeira et al., 2010). To address which tissues affect the behaviour of the chromatophores in *mau* mutants, we expressed the tVE1 variant (i.e. Aqp3aR220Q) fused to sfGFP under the ubiquitous *ef1a* promoter (Fig. 6A) and analyzed the phenotypes arising from mosaic overexpression of the protein in random tissues.

We observed pattern irregularities, the appearance of ectopic melanophores in the light stripe regions, and melanophore and iridophore death when large epidermal clones coincided with large muscle clones (Fig. 6B, top). Neither large muscle clones (Fig. 6B, bottom) nor epidermal clones alone were sufficient to affect the pigment pattern. Additionally, the expression of Aqp3aR220Q in xanthophores did not affect stripe formation (Fig. 6B, bottom), substantiating our transplantation experiment results. No effect on pigment patterning was observed upon overexpression of the wild-type variant of Aqp3a (Fig. 6C). We also created a transgenic line expressing sfGFP-Aqp3a wild type (WT) or R220Q under the epidermis-specific keratin promoters *krtt1c19e* [basal layer of epidermis, which gives rise to all the strata of adult skin (Lee et al., 2014)] and *krt4* [also known as *krt8*, enveloping layer and periderm-specific (Gong et al., 2002)]. Neither of these transgenes had any effect on the trunk patterning, although *Tg(krtt1c19e:sfGFP-Aqp3aR220Q)* fish showed slight defects in fin pigmentation (e.g. the stripes do not reach the margin of the caudal fin). *Tg(krt4:Aqp3aR220Q)* fish occasionally exhibited spotted fins resembling the pigmentation of heterozygous *tVE1* fish and weaker *mau* mutant alleles (Fig. 6D,E). *Tg(krt4:sfGFP-Aqp3aR220Q)* fish have short fins, similar to *aqp3a^tVE1/+^* (Fig. 6F). Although the *krt4* promoter is known to drive expression in the periderm and simple epithelia (Imboden et al., 1997; Gong et al., 2002), we observed weak fluorescence in fin ray segments of post-metamorphic and adult fish (Fig. 6G,H), and this expression is likely to be responsible for the short fin phenotype.

## DISCUSSION

We describe novel mutations in *aqp3a* in zebrafish that result in an aberrant pigmentation pattern as well as short fins. We show that pigment cells are not directly affected, but that the tissue environment is changed by the mutations.

Aqp3a is known to facilitate the transmembrane transport of water and small non-polar solutes such as glycerol, peroxide and urea (Tingaud-Sequeira et al., 2010). The functionality of the pore is determined by six transmembrane domains and two semi-transmembrane loops, which work as a water selectivity filter and exclude ions from the pore (Sui et al., 2001). All four dominant *mau* mutant alleles have amino acid exchanges in the pore surface of the protein. The strongest allele (*tVE1*) replaces an invariant arginine residue of the water selectivity filter with glutamine, while the three weaker alleles affect other conserved residues. Each mutation alters the pore permeability for water and glycerol, which are known cargos for Aqp3 in mammals (Ma et al., 1994; Echevarria et al., 1994; Miller et al., 2010). However, the decrease in water and glycerol transport cannot explain the dominant *mau* phenotype, as the complete loss-of-function mutation reverts the *mau* phenotype to wild type. Our preliminary data suggest that there are alterations in calcium homeostasis in the skin of *mau* mutants (A.E., U.I. and C.N.-V., unpublished). In the three weaker alleles we find ER retention of the mutant proteins, implying that protein folding might be affected in addition to permeability, resulting in reduced amounts of protein reaching the membrane. This might lead to the attenuation of the phenotype compared with *tVE1*.

Mutations in the human gene leading to truncations of AQP3 also have no phenotypic consequences (Roudier et al., 2002). An *Aqp3* null mutation in mice results in a weak phenotype with excess urine production and mild defects in skin thickness and hydration (Ma et al., 2000; Hara-Chikuma and Verkman, 2008). The fact that the *aqp3a* loss-of-function mutation that we generated by CRISPR-Cas9 shows no phenotype might also be due to redundancy between *aqp3a* and *aqp3b*, the two paralogues present in the zebrafish genome (Tingaud-Sequeira et al., 2010).

All three types of chromatophores from *mau* mutants can contribute to a normal pigment pattern in a wild-type environment. The development of pigment cells in *mau* shows that all three cell types initially appear normal, but later fail to form a proper striped pattern: the boundaries between the light and dark stripes are not well defined and the stripes break up into spots.

Interactions between chromatophores are required for their proliferation, survival and normal behaviour, and are essential for pigment pattern formation (Frohnhöfer et al., 2013; Singh and Nüsslein-Volhard, 2015; Parichy and Spiewak, 2015; Walderich et al., 2016; Mahalwar et al., 2016). In *mau* mutants the most severely affected cells are the larval xanthophores, which lose contacts and mostly disappear from the flanks of the fish shortly before metamorphosis. Larval xanthophores give rise to metamorphic xanthophores, which in *mau* are present in lower numbers and with an altered morphology. As xanthophores are required for shaping the dark stripes (Parichy and Turner, 2003; Frohnhöfer et al., 2013; Iwashita et al., 2006), the abnormal distribution of melanophores that we find in *mau* could be the result of impaired xanthophore-melanophore communication. In *pfeffer* (*csf1ra*) mutants, which completely lack xanthophores, the melanophores are scattered outside of the dark stripe region and their number is reduced (Parichy et al., 2000a; Parichy and Turner, 2003). However, unlike *pfeffer* mutants, *mau* mutants have the normal number of melanophores, suggesting that there are enough xanthophores to support the survival of melanophores. It is conceivable that the abnormal positioning of melanophores during stripe formation in *mau* mutants prevents iridophores from aggregating and forming a regular second light stripe, ultimately yielding a broken striped pattern. Consistent with this, *pfeffer* and *mau* double mutants are similar to *pfeffer* mutants in terms of pigment pattern (data not shown).

Thus far, no mutants have been described that resemble *mau* in terms of larval xanthophore development. However, temporal inactivation of Csf1ra during the early stages of xanthophore development, using a temperature-sensitive *csf1ra* allele, and then reactivation during metamorphosis leads to a scattering of melanophores across the flank. Interestingly, fish carrying a temperature-sensitive allele and a null allele for *csf1ra* show stripe breakages (Parichy and Turner, 2003) resembling the pattern that we observe in heterozygous *tVE1* fish. Nonetheless, the *mau* phenotype is stronger than phenotypes caused by temporary loss of xanthophores and of Csf1ra signalling (Parichy and Turner, 2003), implying that the cellular behaviour of the other two pigment cell types (melanophores and iridophores) is also affected in the *mau* mutant environment.

The failure of wild-type pigment cells to organize into stripes when transplanted into *mau* mutants suggests that they receive the wrong cues from non-pigment cells or the extracellular environment. As Aqp3a is a pore protein and the mutant variants act in a dose-dependent manner, it seems likely that they affect the pigment cells and fin growth locally. Skin pigment cells in zebrafish are located in the hypodermis. They are overlaid by layers of epidermal keratinocytes and loosely packed dermal fibroblasts (Hirata et al., 2003, 2005). Beneath them are thin layers of slow and fast muscle bundles (van Raamsdonk et al., 1982). We found that expression of the tVE1 variant (Aqp3aR220Q) in large areas of both the epidermis and the underlying muscles leads to some stripe disruption and the appearance of ectopic melanophores within the light stripe region, resembling the *mau* phenotype. Smaller patches of cells do not yield such a phenotype, nor do clones that are restricted to muscle or epidermal cells only, suggesting that a minimal number of cells in the adjacent tissues is needed to affect the behaviour of the chromatophores.

Pigmentation in zebrafish presents an attractive system with which to study the cellular basis of pattern formation processes *in vivo*. The cell-autonomous interactions between the three types of chromatophores, which are necessary to produce the stripes, occur in a tissue-specific context. Our study emphasizes the role that the cellular environment plays in pigment cell behaviour and pattern formation, and the role that it might play in the immense diversification of pigment patterns in fish and other ectothermic vertebrates.

## MATERIALS AND METHODS

### Zebrafish maintenance

Fish were maintained and bred as described by Nüsslein-Volhard and Dahm (2002). The following genotypes were used: TU and WIK wild-type strains; *nacre^w2^* (Lister et al., 1999), *pfeffer^tm236b^* (Odenthal et al., 1996), *transparent^b6^* (Krauss et al., 2013), *Tg(TDL358:GFP)* (Levesque et al., 2013), *Tg(sox10:mRFP)* [M. Levesque (Singh et al., 2014)] and *Tg(fms:GAL4),Tg(UAS:E1b:nfsB.mCherry)*, which is referred to in the text as *Tg(fms:Gal4; UAS:mCherry)* (Gray et al., 2011).

All animal experiments were performed in accordance with the guidelines of the State of Baden-Württemberg, Germany, and approved by the Regierungspräsidium Tübingen (35/9185.46-5, 35/9185.82-7, 35/9185.81-5).

### Genetic mapping

Heterozygous offspring of a cross between *tWGU2* and WIK were used for meiotic mapping as described (Nüsslein-Volhard and Dahm, 2002). The mutation was mapped to the region between microsatellite markers z9568 (36.7 cM) and z13641 (41.4 cM) on chromosome 5.

For next-generation sequencing, a genomic DNA library was produced from a single homozygous *tVE1* *mau* mutant and WIK sibling fish using the NEBNext DNA Library Kit (NEB) and sequenced on a HiSeq 2000 Sequencing System (Illumina). The sequences were assembled with CLC Genomics Workbench (Qiagen), using Zv9 (GCA_000002035.2) as a template.

### Generating the *aqp3a* loss-of-function allele

CRISPR-Cas9 was used to generate *aqp3a* loss-of-function alleles as described (Hwang et al., 2013). The following oligonucleotides were cloned into pDR274 to create the vector for *in vitro* transcription of the gRNA: Aqp3a_KO_1, 5′-TAGGGGGGTGAGATATTTGCTT-3′; and Aqp3a_KO_2, 5′-AAACAAGCAAATATCTCACCCC-3′. The vector was linearized with *Dra*I, and the 285 bp fragment was gel purified and used as template for *in vitro* transcription with the Megascript Kit (Ambion). Cas9 mRNA was transcribed from the linearized plasmid pMLM3613 using the mMessage mMachine Kit (Ambion). Embryos from an incross of *aqp3a^+/tVE1^* were injected at the 1-cell stage with 2-5 nl 12 ng/µl gRNA and 120 ng/µl Cas9 mRNA. Juvenile fish were genotyped using primers Aqp3a_gen_fw (5′-ATAGGCACAGCATCCCTG-3′) and Aqp3a_gen_rev (5′-GGAATTTGTCGCTAGCCTGT-3′) and *aqp3a^tVE1/tVE1^* mutants were identified. Indels of 1 bp and 4 bp were identified and used to establish the loss-of-function stock.

### Cell culture, transfection and immunostaining

HeLa cells (ATCC CCL-2) were cultured in DMEM (Sigma-Aldrich) containing 10% fetal calf serum (FCS), 2 mM glutamine, 100 U/ml penicillin and 100 µg/ml streptomycin. Transfection of Strep-tagged Aqp3a (KY379154, KY379155, KY379156, KY379157, KY379158) was achieved using Lipofectamine 2000 (Invitrogen) according to the manufacturer's protocol. Twenty-four hours after transfection, the cells were fixed in 4% paraformaldehyde, washed with 0.1% Triton X-100 in PBS (PBST), blocked in 10% normal goat serum (NGS) in PBST and incubated with anti-strep II antibody (1:200; IBA, 2-1507-001). For subsequent staining of calnexin, cells were permeabilized with methanol at −20°C, blocked with 5% NGS in PBST, and incubated overnight at 4°C with anti-calnexin antibodies (1:50; C5C9, Cell Signaling Technology, #2679S). Secondary antibodies were anti-mouse Alexa 647 and anti-rabbit Alexa 488 (1:300; Molecular Probes, A21236 and A11008).

### Transgenic lines, transient and mosaic expression

The coding sequences of wild-type and mutant Aqp3a were amplified from the cDNA of fish using primers: AE1_fw, 5′-ATCAGCGGCCGCATGGGTTGGCAGAAAAGCG-3′; and AE2_rev, 5′-GATGAATTCTCATTCCTTGCTGGCGAC-3′.

We expressed sfGFP-Aqp3a variants transiently in zebrafish embryos to assess protein localization. sfGFP was fused in-frame to the coding sequence of Aqp3a and cloned into pCS2+ (the resulting constructs were KU645301, KU645302, KU645303, KU645304, KU645305). The linearized plasmids were transcribed *in vitro* using the SP6 mMessage Machine Kit (Ambion), and 100 ng/µl of mRNA was injected into 1-cell stage embryos.

For ubiquitous mosaic expression (Perathoner et al., 2014), as well as epidermis-specific expression of Aqp3a WT and Aqp3aR220Q under the *krt4* (Gong et al., 2002) and *krtt1c19* (Lee et al., 2014) promoters, the constructs KU645306, KU645307, KU645308, KY379161, KY379160 and KY379159 were injected into 1-cell stage embryos as a mixture of circular DNA (10-20 ng/µl) and Tol2 mRNA (25 ng/µl).

### Quantification of Aqp3a expression

Embryos from an *aqp3a*^+/−^ incross were genotyped using primers Aqp3a_del_fw (5′-TGTTACATTTGCTCTCTG-3′) and Aqp3a_del_rev (5′-TAACCATCCCCATGTCCCAG-3′) to identify *aqp3a*^−/−^ and wild-type siblings. RNA was extracted with TRIzol (Life Technologies) and cDNA was produced using SuperScript II reverse transcriptase (Thermo Fisher Scientific). *aqp3a* transcripts were amplified using Aqp3a_gen_fw and AE2_rev for 35 cycles. *actb1* transcripts were amplified using primers actb1_fw (5′-ACTGGGATGACATGGAGAAGAT-3′) and actb1_rev (5′-GTGTTGAAGGTCTCGAACATGA-3′) for 28 cycles. The relative abundance of the transcripts was quantified from the gel using Fiji (Schindelin et al., 2012) and averaged between biological replicates.

### Transplantations

Chimeric fish were produced as described (Nüsslein-Volhard and Dahm, 2002) by transplanting 20-40 blastomeres from donor to host embryos at the 1000-cell blastula stage.

### Image acquisition and processing

Fish were anaesthetized for imaging with 0.004% MS-222 (Sigma). Embryos at 1-5 dpf were embedded in 0.5% low-melting agarose. Images of embryos and metamorphic fish were acquired on a Zeiss LSM 780 NLO confocal microscope and processed with Fiji (Schindelin et al., 2012) and ZEN 2012 (Zeiss). Confocal *z*-stacks are shown as maximum intensity projections. Follow-up of pigment cell development was by time-lapse imaging of metamorphic fish, imaging the mid-body area once a day. Adult fish were anaesthetized as above and photographed with a Canon EOS 5D MarkII camera and a Macro 100 objective.

### Pigment cell counts

Pigment cell counts were performed using maximum intensity projection images obtained from confocal *z*-stacks using the Fiji Cell Counter plugin. We counted pigment cells from two or three mid-body segments. For counts of melanophores, fish were treated with epinephrine, and only melanophores on the flank of the fish that contribute to the stripe pattern were taken into consideration. The xanthophores were counted in *Tg(fms:GAL4),Tg(UAS:E1b:nfsB-mCherry)* double-transgenic fish from 9 to 21 dpf*.* The iridophores were counted in *Tg(TDL358:GFP)* (Levesque et al., 2013) and *Tg(sox10:mRFP)* fish at SL 6-6.5 mm and 6.5-7.5 mm. The fish were imaged daily from 23 to 26 dpf. The number of iridophores per segment was normalized to the number of iridophores in the same segment at 23 dpf.

### Functional expression in *Xenopus* oocytes

Wild-type and mutant cDNAs were cloned into pT7Ts, and cRNAs for microinjection were synthesized with T7 RNA polymerase (Roche). Microinjections of stage V and VI oocytes were performed as described by Deen et al. (1994). Oocytes were transferred to modified Barth's medium (MBS) [88 mM NaCl, 1 mM KCl, 2.4 mM NaHCO_3_, 0.82 mM MgSO_4_, 0.33 mM Ca(NO_3_)_2_, 0.41 mM CaCl_2_, 10 mM HEPES, 25 mg/ml gentamycin, pH 7.5], and injected with 50 nl distilled water (negative control) or 2 ng cRNA in 50 nl water.

At 24 h, the oocytes were manually defolliculated, and maintained in MBS at 18°C. To determine the osmotic water permeability (*P*_f_), the oocytes were transferred at 48 h to 10-fold diluted MBS (20 mOsm). Oocyte swelling was tracked by video microscopy using serial images at 2 s intervals for 20 s. *P*_f_ values were calculated taking into account the timecourse changes in relative oocyte volume [d(V/V_0_)/dt], the partial molar volume of water (V_W_=18 cm^3^/mol), and the oocyte surface area (S) using the formula V_0_[d(V/V_0_)/dt]/[SV_W_(Osm_in_−Osm_out_)]. The surface area of the oocyte was considered to be nine times the apparent area because of membrane folding (Zampighi et al., 1995).

Glycerol permeability (*P*_gly_) was also determined volumetrically in isotonic MBS, where NaCl was replaced by 160 mM solute. Oocyte swelling was measured by video microscopy, using serial images at 5 s intervals for 1 min. *P*_gly_ was calculated from oocyte swelling using the formula [d(V/V_0_)dt]/(S/V_0_) (Verkman and Ives, 1986). The uptake of H_2_O_2_ was determined using the ROS-sensitive, cell-permeable fluorescent dye 5-(and-6)-chloromethyl-2′,7′-dichlorodihydrofluorescein diacetate, acetyl ester (CM-H_2_DCFDA; C6827, Life Technologies), following a method described by Chauvigné et al. (2015). Oocytes were incubated with isotonic MBS plus 0.5% DMSO and 200 µM CM-H_2_DCFDA for 1 h at 18°C. After washing in MBS, the oocytes were exposed to 100 µM H_2_O_2_ in MBS for 30 min at 18°C. The CM-H_2_DCFDA fluorescence of each well (oocyte plus MBS) was measured at excitation and emission wavelengths of 495 nm and 525 nm, respectively, using a multiwell plate reader (InfiniteM200, Tecan). We corrected the background to remove nonspecific signals from MBS and from oocytes not exposed to the dye.

To study the relative expression of Aqp3a mutants with respect to wild type, constructs carrying the human influenza hemagglutinin (HA) tag at the N-terminus (nucleotide sequence: 5′-TACCCATACGATGTTCCAGATTACGCT-3′ or 5′-TATCCATATGATGTTCCAGATTATGCT-3′) were PCR amplified and 15 ng of the corresponding cRNAs was injected into *Xenopus* oocytes. For western blotting, total membranes were isolated from groups of ten oocytes as previously described (Kamsteeg and Deen, 2001). Protein samples equivalent to the total membrane extract of two oocytes per lane were denatured at 95°C for 10 min in Laemmli buffer, electrophoresed on a 12% polyacrylamide SDS gel, and then blotted onto nitrocellulose membranes (Bio-Rad). Membranes were blocked for 1 h with TBST (20 mM Tris, 140 mM NaCl, 0.1% Tween, pH 8) containing 5% nonfat dried milk (Sigma-Aldrich), and then incubated with 1:1000 HA polyclonal rabbit antibody (PA1-985, Life Technologies) in TBST with 5% nonfat dried milk at 4°C overnight. Horseradish peroxidase-coupled goat anti-rabbit IgG antibody (Santa Cruz Biotechnology, sc-2004) was used at 1:5000. Reactive protein bands were detected using enhanced chemiluminescence western HRP substrate (ECL, Millipore).

### Homology modelling

HHpred (Söding et al., 2005) was used to identify homologues of known structure. HHpred searches were conducted in default settings against the protein data bank (PDB) (Berman et al., 2000), as available on 1st February 2016, and clustered at 70% pairwise sequence identity. Searches of the *D. rerio* Aqp3a sequence retrieved glycerol uptake facilitator protein from *E. coli* (PDB identifier 1LDF) and aqua-glyceroporin from *P. falciparum* (PDB identifier 3C02) as the closest homologues. These structures served as templates for generating homology models with Modeller (Sali and Blundell, 1993).

### Statistical analysis

Statistical analysis was performed using InStat (GraphPad Software).
